# Inflammatory Long Pentraxin 3 is Associated with Leukocyte Telomere Length in Night-Shift Workers

**DOI:** 10.3389/fimmu.2017.00516

**Published:** 2017-05-09

**Authors:** Sofia Pavanello, Mariarita Stendardo, Giuseppe Mastrangelo, Melissa Bonci, Barbara Bottazzi, Manuela Campisi, Marco Nardini, Roberto Leone, Alberto Mantovani, Piera Boschetto

**Affiliations:** ^1^Occupational Medicine, Department of Cardiac, Thoracic and Vascular Sciences, University of Padova, Padova, Italy; ^2^Department of Medical Sciences, University of Ferrara, Ferrara, Italy; ^3^Laboratory of Research in Immunology and Inflammation, Humanitas Clinical and Research Center, Rozzano, Milan, Italy; ^4^Department of Prevention and Protection, University-Hospital and Public Health Service of Ferrara, Ferrara, Italy; ^5^Humanitas University, Rozzano, Milan, Italy

**Keywords:** inflammation, aging, premature, telomere, pentraxin 3, circadian rhythm, genetic instability, night-shift work

## Abstract

Aging is an emerging worldwide threat to public health, even in the workplace, as it links with risk of illness and death. Bewildered inflammatory responses and stressful conditions associate with age-related disorders. Additionally, circadian rhythm disruption, a critical health issue in night-shift workers, correlates with premature aging. We investigated the hypothesis of a link between altered inflammatory response, detected by plasmatic long pentraxin 3 (PTX3), and biological aging, measured by leukocyte telomere length (LTL), attrition, and possibly induced by night-shift work. Within the framework of a cross-sectional study, such possible relationships were appraised by simultaneous equation model (SEM) technique among day and night-shift hospital workers. PTX3 levels, modulated by several aging conditions [i.e., body mass index (BMI) (beta = −0.22; *p* = 0.022), C-reactive protein (CRP) (beta = −0.07; *p* = 0.000), and cardiovascular diseases with hypertension included (CVD) (beta = −0.12; *p* = 0.000)], positively associate with LTL (coefficient = 0.15; *p* = 0.033). LTL, in turn is reduced by CVD (beta = −0.15; *p* = 0.000), binge drinking (beta = −0.10; *p* = 0.004), and CRP (beta = −0.05; *p* = 0.026). On the other hand, night-shift work, found to be remarkably free from aging risk factors [i.e., age (beta = −0.13; *p* = 0.017), BMI (beta = −0.17; *p* = 0.030), CVD (beta = −0.14; *p* = 0.000), and binge drinking (beta = −0.13; *p* = 0.000)], does associate almost significantly with reversed PTX3 (coefficient = −0.09; *p* = 0.089) and even with CRP (beta = 0.17; *p* = 0.000). In conclusion, the SEM analysis indicates that PTX3 is positively linked to LTL. The finding suggests a possible new role of this long pentraxin that, by orchestrating an efficient governance of inflammatory processes, may protect telomere from attrition, ensuring therefore the genetic stability of cells. The higher CRP levels among night-shift workers suggest that night-shift work is associated with increased systemic inflammation. This would make nocturnal workers more susceptible to premature aging.

## Introduction

Aging is an emerging worldwide threat to public health even in the workplace (1). The aging of the general population inescapably influences the composition of the workforce (CDC, http://www.cdc.gov/nchs/fastats/deaths.htm). In most industrialized countries, including Italy, the proportion of workers aged ≥45 years has increased by as much as 50% in the past 20 years and such increase is expected to continue in the next 20 years (2). As the population ages, the global burden of disease and disability is rising, with a negative impact on the “employability” of “elderly” workers. About 50% of those between 55 and 59 years, in fact, suffer from at least one chronic disease (2).

Aging is an individual, natural, inexorable, and biologically complex process during which a decrease in the body’s ability progressively appears to respond appropriately to internal and/or external stressors. This results in an increased risk of illness and death (3). Some individuals have a more rapid physiological senescence than others (4). Consequently, age, when measured chronologically, may not be a reliable indicator of the body’s rate of decline or physiological breakdown, but rather, serves only as an estimation of the rate of aging (5).

All the cells in our body have a biological aging clock in telomeres, the repetitive functional complexes of DNA/protein at the ends of chromosomes. Telomeres preserve DNA integrity that in their absence would be gradually lost with each cell division (6). A specific enzyme, telomerase is involved in telomere synthesis after mitosis, but is active only in progenitor cells and in certain diseases (7). The telomeres therefore shorten progressively with each division of somatic cells and their length measured in peripheral blood lymphocytes (LTL) is considered an indicator of biological age (8). Altered inflammatory responses (9) and exposure to environmental (10) and occupational agents (11), favoring the oxidative stress (12) can accelerate the physiological telomere erosion increasing the risk of age-related disease, i.e., chronic degenerative disease, including cancer (13).

Long pentraxin 3 (PTX3) is an acute phase protein that belongs to the same family of C-reactive protein (CRP), prototype of the short pentraxins (14). PTX3 is produced in response to primary inflammatory stimuli or microbial recognition and exerts non-redundant roles in innate immunity and in regulation of inflammation. Several observations point to a safeguarding role of PTX3 against cardiovascular disease (CD) (15) and tumor (16), acting through a functional/efficient tuning of the inflammation process and representing an extreme attempt of the body to limit an excessive inflammatory response (17).

Circadian rhythm disruption has become an emerging health issue being associated with premature aging and early onset of chronic conditions such as obesity, CD, and metabolic diseases (18, 19). By disrupting the human circadian rhythm, night-shift work is recognized as affecting several age-related disorders (20, 21). However, the mechanisms of this association are not well understood. With this respect, inflammation and the consequent biological aging is a plausible pathophysiological mechanism through which night-shift work may influence the observed risk of diseases (22). Biomarkers of biological aging, such as LTL, and of inflammation, such as PTX3, have never been examined in night-shift workers.

Aim of this study was, therefore, to explore the hypothesis of a link between inflammation, evaluated by PTX3 plasma levels, and biological aging, measured by LTL, in relation to night-shift work. This association was evaluated within the framework of a cross-sectional study conducted among day and night-shift workers of the five territorial hospitals in Ferrara, taking into account the occupational, physiological, pathological, and pharmacological history of each participant. Such complex interrelationships were appraised by the technique of simultaneous equation model (SEM). This estimation procedure uses a set of simultaneous regression equations and yields coefficient estimators more efficiently than single-equation estimators. It has recently been implemented in the program of simultaneous equation model of STATA (version 14).

## Materials and Methods

### Study Design

A cross-sectional study was carried out involving the following steps: (a) nurses, working at the five territorial hospitals of the Ferrara area, were enrolled (between March 2015 and July 2015) during their periodic check-ups at the Preventive Medicine Surgery. Participants were identified by scrutinizing the hospital’s occupational medicine service’s computerized records. The inclusion criteria were: >18 and <65 years of age. (b) Subjects were informed of the purpose of the study by trained interviewers and asked to sign an informed consent form. (c) Study participants underwent a physical examination, an interview with structured questionnaires administered by trained interviewers and fasting blood sample collection for laboratory tests (i.e., basic biochemistry, CRP, telomere length, and PTX3 analyses) at the end of the shift work. The local Ethics Committee (School of Medicine, University of Ferrara) approved the study protocol (code number 140792). All subjects gave written informed consent and the study was conducted in accordance with the Declaration of Helsinki.

### Subjects

Study population consisted of 84 day workers and 71 night-shift workers. A night-shift was defined as a work-shift schedule that included a night shift (from 8 p.m. to 6 a.m.). From a total of 342, 186 nurses gave their consent, but only 155 agreed to participate in the protocol definitively. Characteristics of the study subjects (physical examination, information acquired through questionnaires and laboratory tests) are shown in Tables 1 and 2.

**Table 1:** **Interval variables in 84 day workers and 71 night-shift workers (mean ± SD) and *p*-values of the one-way ANOVA comparing the two groups**.

Variables	Day workers	Night-shift workers	*p*-Value
Age (years)	48.9 ± 6.3	46.7 ± 5.3	**0.018**
Length of employment (years)	25.9 ± 7.4	22.6 ± 6.3	**0.004**
Length in the current job (years)	12.0 ± 8.8	22.0 ± 6.7	**0.001**
Work ability index (WAI score)	37.0 ± 4.8	38.3 ± 5.1	0.123
Education (years)	15.0 ± 1.6	15 ± 1.5	0.887
Body mass index (kg/m^2^)	26.2 ± 5.2	25.5 ± 5.2	0.468
Waist (cm)	97.5 ± 12.0	96.3 ± 12.9	0.549
Systolic pressure (mm Hg)	117.5 ± 15.6	115.5 ± 13.2	0.398
Diastolic pressure (mm Hg)	75.3 ± 9.8	74.3 ± 9.8	0.529
Mother age (years)	27.4 ± 6.9	28.3 ± 5.8	0.385
Father age (years)	30.7 ± 6.3	31.3 ± 6.4	0.517
Pack-years [(cigarettes/20) × years]	7.5 ± 10.0	7.3 ± 10.6	0.886
Drinking (age at start, years)	13.4 ± 11.7	13.8 ± 11.3	0.835
Alcohol (daily intake last year)	0.2 ± 0.3	0.2 ± 0.4	0.502
Sport (IPAQ score)	2.7 ± 1.9	3.9 ± 3.3	**0.003**
Leukocytes (10^3^/ml)	6.6 ± 1.7	7.2 ± 1.9	0.065
Blood red cells (10^3^/ml)	4.6 ± 0.5	4.6 ± 0.4	0.423
Hemoglobin (g/dl)	13.2 ± 1.4	13.0 ± 1.4	0.498
Platelet count (10^3^/ml)	275.8 ± 64.6	280.4 ± 68.5	0.670
Neutrophils (10^3^/ml)	3.5 ± 1.3	3.6 ± 1.1	0.391
Lymphocytes (10^3^/ml)	2.4 ± 0.6	2.7 ± 0.9	**0.020**
Monocytes (10^3^/ml)	0.5 ± 0.1	0.6 ± 0.1	**0.009**
Eosinophils (10^3^/ml)	0.2 ± 0.1	0.2 ± 0.1	0.943
Basophils (10^3^/ml)	0.03 ± 0.01	0.03 ± 0.01	0.397
Glycemia (mg/dl)	85.1 ± 12.8	81.0 ± 14.5	0.067
Cholesterol (mg/dl)	204.8 ± 37.8	205.4 ± 42.5	0.925
Triglycerides (mg/dl)	91.3 ± 43.3	100.3 ± 53.6	0.254
Low-density lipoprotein (mg/dl)	118.8 ± 33.1	119.0 ± 38.0	0.968
High-density lipoprotein (mg/dl)	67.4 ± 17.2	68.5 ± 17.9	0.695
C-reactive protein (mg/ml)	0.3 ± 0.2	0.4 ± 0.2	0.144
Glycated hemoglobin (mmol/mol)	36.3 ± 6.6	35.8 ± 7.8	0.670
Pentraxin 3 (ng/ml)	3.9 ± 3.3	3.6 ± 1.7	0.412
Leukocyte telomere length (T/S)	1.1 ± 0.4	1.3 ± 0.5	**0.025**

*Bold character is displayed only for significant values*.

**Table 2:** **Distribution of categorical variables in 84 day workers and 71 night-shift workers with *p*-values of the chi-square test comparing the two groups**.

Variables	Classes	Day workers		Night-shift workers		*p*-Value
		Number	Percent	Number	Percent	
Sex^a^	Males	7	8.3	8	11.3	0.538
Nights at work	0	84	100.0	0	0.0	**0.001**
	2–4			22	30.9	
	5+			49	69.0	
Work injury^a^	1 or more events	26	30.0	21	29.5	0.853
Smoking	Non-smokers	37	44.1	35	49.3	0.535
	Ex smokers	23	27.3	14	19.7	
	Smokers	24	28.5	22	30.9	
Drink^a^	Drinkers	53	63.1	46	64.7	0.827
Binge	None	75	89.2	67	94.3	0.439
	4	8	9.5	3	4.2	
	≥5	1	1.2	1	1.4	
Chronic disease^a^	1 or more events	74	88.1	48	67.6	**0.002**
Charlson index^b^	≥1	4	78.7	4	83.1	0.374
Musculoskeletal disease^a^	1 or more events	57	67.8	37	52.1	**0.046**
Spinal disk hernia^a^	1 or more events	49	58.3	29	40.8	**0.030**
Cardiovascular disease^c^	1 or more events	38	42.2	15	21.1	**0.002**
Gastrointestinal disease^a^	1 or more events	21	25.0	17	23.9	0.879
Endocrine disease^a^	1 or more events	24	28.5	18	25.3	0.653
Diabetes^a^	Yes	4	4.7	3	4.2	0.873
Respiratory^a^	Yes	8	9.5	7	9.8	0.944
Tumors^a^	Yes	6	7.1	5	7.0	0.981

*Bold character is displayed only for significant values*.

*^a^Dichotomous variable; table reports the individuals belonging to the class coded as 1; the remaining subjects may be obtained by subtraction*.

*^b^From the Charlson index were excluded conditions (i.e., in our study population specifically were diabetes, tumors, and/or respiratory diseases) that significantly impact on inflammatory response*.

*^c^Cardiovascular disease includes arterial hypertension disease*.

### Physical Examination

Waist circumference, height and weight, and the body mass index (BMI: body weight in kilograms divided by height in meters squared) were collected. Systolic and diastolic arterial pressures were the mean of three values measured at 10-min interval.

### Information Acquired through Questionnaires

Structured questionnaires were administered during interviews to elicit information on: demographics (age, sex, marital status) and other personal information (mother/father age at birth, years of education); occupation [job title; hospital department; total years worked; years spent in the current job; shift work (work was considered scheduled in day shift from 6 a.m. to 2 p.m., afternoon shift from 2 p.m. to 8 p.m. and night-shift from 8 p.m. to 6 a.m.); and frequency of night shifts/month; job energy requirement (expressed as metabolic equivalent) at work; work injury; work ability index (WAI)]. The latter has proved a valuable tool to identify any imbalances between what is required (performance requirements) and what you are able to give (individual potential) (23) and consisted of a seven-part self-assessment: current ability, work ability in relation to physical and mental demands of the job, reported diagnosed diseases, estimated impairment due to health status, sick leave over the previous 12 months, self-prognosis of work ability in the following 2 years, and mental resources of the individual. WAI ranged from 7 to 49 points; four categories have been suggested to describe WAI levels: poor (7–27), moderate (28–36), good (37–43), and excellent (44–49). Smoking history (current active smokers, former-smokers, never smokers) and pack-years (cumulative lifelong consumption of tobacco: cigarettes day/20× years) were also recorded. Habitual alcohol consumption was a dichotomous variable (yes/no), while alcohol intake was expressed in units of drink/day, each unit being approximately 10–12 g alcohol intake. Binge was defined as >4 drink-units/day (more than 40 g alcohol/day) (10). Alcohol intake in the last year was also calculated. Physical activity in leisure time was estimated according to the International Physical Activity Questionnaire (IPAQ score); finally, medically relevant complaints were arranged in groups to include cardiovascular disease (arterial hypertension included), musculoskeletal disorder, spinal disk hernia, gastrointestinal disease, endocrine disease, diabetes, respiratory disease, and tumors. The Charlson comorbidity index (24), a method of predicting mortality by classifying or weighting comorbid conditions (comorbidities), was calculated, excluding diabetes, tumors and/or respiratory diseases, and other inflammatory conditions.

### Laboratory Tests

#### Basic Biochemistry

Data of basic biochemistry included number of blood red cells, platelets, lymphocytes, monocytes, neutrophils, basophils, eosinophils, and white cells, hematocrit, hemoglobin, glycated hemoglobin, blood glucose, triglycerides, cholesterol, low-density lipoprotein, high-density lipoprotein, and CRP.

#### LTL Analyses

Leukocyte telomere length was measured after DNA extraction from whole blood by Genomic DNA Purification Kit Protocol (Wizard^®^, Promega, Italy) and quantification, using the Quantus™ Fluorometer (Promega, Italy). DNA was available for all study subjects. LTL DNA was measured by the real-time quantitative PCR method developed by Cawthon (25) and described previously (10, 11). This method measures the relative TL in genomic DNA by determining the ratio of telomere repeat copy number (T) to single-copy gene (S) (T:S ratio) in experimental samples relative to the T/S ratio of a reference pooled sample (10, 11). The single-copy gene used in this study was human β-globin (hbg). A “seven-point” standard curve was generated from a serially diluted DNA pool (obtained from 50 DNA samples randomly selected from the DNA samples tested in the present study), ranging from 20 to 0.31 ng in each plate, in order to determine relative quantities of T and S (in nanograms). All samples and standards were run in triplicate and the average of the 3 T/S ratio measurements was used in the statistical analyses. The PCR runs were conducted in triplicate on a SteponePlus Real-Time PCR System (Applied Biosystems). After PCR amplification, the specificity of the product was confirmed by dissociation curve analysis. To test the reproducibility of telomere length measurements, we amplified telomere (T) and hbg (S) in 15 samples replicated 3 times on 3 different days. The within-sample CV for the average T/S ratio over the three consecutive days was 8.5%, which was similar to the CV reported for the original protocol (25).

#### PTX3 Analyses

Plasma levels of PTX3 were measured by in-house sandwich ELISA as previously described (26). Detection limit was 100 pg/ml. No cross-reaction with human CRP or serum amyloid P component was observed for antibodies used to detect PTX3. The concentration of PTX3 was determined by comparison with a standard curve made with recombinant human PTX3 (range from 75 pg/ml to 2.4 ng/ml).

### Statistical Analysis

One-way ANOVA and chi-square test were used to compare interval and categorical variables in the two groups of 84 day workers and 71 night-shift workers (Tables 1 and 2).

A hypothesis-driven analysis was performed with simultaneous equation modeling (SEM) analysis, where PTX3, LTL, and night-shift work were the Y variables (also termed “endogenous” variables) of three simultaneous regression equations. Since the same regressors (see below) were specified for each equation, the analysis was carried out using a simultaneous equation modeling program (SEM in Stata commands) rather than the Stata command which registers seemingly unrelated regression (sureg). SEM was used since it provides robust standard errors whereas sureg does not. Moreover, using the method “adf” (asymptotic distribution free) to obtain fitted parameters, SEM did not make assumptions on joint normality of all the variables and allowed using the variables (interval or categorical) as given. Finally, using the SEM option “stand,” the effect of each predictor can be expressed as standardized (or beta) coefficients that make comparisons easily by ignoring the independent variable’s scale of units. The other side of the coin is that it can be devilishly difficult for SEM to identify the best set of predictor variables. Often, SEM did not converge, reiterating while producing little improvement in the log-likelihood value. Since the error messages were generally not helpful, to solve the convergence problems, we simplified the model using a set of predictors (also called exogenous variables) not highly correlated to each other. Therefore, the regressors of the three equations above were the following: age, sex, BMI, waist, WAI, pack-years (PY), binge, history of cardiovascular diseases and arterial hypertension (CVD), protein-c-reactive, eosinophils, basophils, and monocytes. We used two SEM’s goodness-of-fit statistics: (1) the chi-square test for “model versus saturated” (the saturated model is the model that fits the covariances perfectly) and (2) the stability index obtained from the analysis of simultaneous equation systems.

## Results

### Baseline Characteristics of the Study Population

In Table 1, night-shift workers compared to day workers showed younger age (*p* = 0.018), lower length of total employment (*p* = 0.004), more years spent in the current job (*p* = 0.001), and they were physically more active according to the IPAQ (*p* = 0.003). All these characteristics are reflected by a significant longer mean LTL (*p* = 0.025), and a higher number of lymphocytes (*p* = 0.020) and monocytes (*p* = 0.009). WAI was quite a bit higher in night-shift workers but the difference was not significant. Table 2 shows that in both groups more than 90% of nurses were females, a feature in line with this type of work. In particular, night shifts were never performed by day workers (*p* = 0.001). Compared to the elder day workers, night-shift workers reported lesser percentages of chronic diseases (*p* = 0.002), musculoskeletal diseases (*p* = 0.046), spinal disk hernia (*p* = 0.030), and CVD (*p* = 0.002). All the other characteristics, including PTX3 plasma levels, smoking, and drinking history, were equally distributed between day and night-shift workers and were within the normal range for healthy subjects.

### Outcomes

Table 3 displays the results of the simultaneous analysis of three regression equations with the Stata program SEM. The results are shown in three groups of columns and three groups of rows. Each group of column is an equation whose endogenous variable is PTX3, telomere length, or night-shift work, as shown in the column headings. The three groups of lines are the following:

- panel A (structural equations) that includes one exogenous variable per line with the corresponding beta coefficients, 95% confidence intervals, and *p*-values; the “minus” sign of beta coefficient shows an inverse relationship;
- panel B (variances) that gives the “error term” of each equation, which is used to estimate the percent of variation explained by the regression (=1−error)%;
- panel C (covariances) that measures the extent to which two equations are related through the correlation in their errors (see above). If, say, error 1 and error 2 have a contemporaneous cross-equation correlation they influence each other contemporaneously, then a SEM program is required to regress.

**Table 3:** **SEM results**.

	Endogenous	PTX3		LTL		Night-shift work	
Exogenous							
			
		Coef (95% CI)	*p*-Value	Coef (95% CI)	*p*-Value	Coef (95% CI)	*p*-Value
(A) Structural equations	Age	−0.10 (−0.21; 0.01)	0.087	−0.05 (−0.14; 0.04)	0.256	**−0.13 (**−**0.24;** −**0.02)**	**0.017**
	Sex	−**0.13 (**−**0.18;** −**0.07)**	**0.000**	0.01 (−0.04; 0.06)	0.726	**0.10 (0.01; 0.18)**	**0.021**
	BMI	−**0.22 (**−**0.40;** −**0.03)**	**0.022**	−0.02 (−0.17; 0.13)	0.790	−**0.17 (**−**0.33;** −**0.02)**	**0.030**
	Waist	0.10 (−0.10; 0.29)	0.321	0.03 (−0.13; 0.18)	0.737	0.11 (−0.06; 0.27)	0.200
	WAI	−0.06 (−0.14; 0.01)	0.098	0.06 (−0.03; 0.16)	0.197	**0.10 (0.00; 0.21)**	**0.046**
	Py	**0.07 (**−**0.00; 0.13)**	**0.061**	0.04 (−0.05; 0.13)	0.409	0.04 (−0.05; 0.13)	0.369
	Binge	−0.05 (−0.11; 0.02)	0.165	**−0.10 (**−**0.16;** −**0.03)**	**0.004**	−**0.13 (**−**0.20;** −**0.06)**	**0.000**
	CVD	−**0.12 (**−**0.17;** −**0.06)**	**0.000**	**−0.15 (**−**0.22;** −**0.08)**	**0.000**	−**0.14 (**−**0.22;** −**0.06)**	**0.000**
	CRP	−**0.07 (**−**0.11;** −**0.03)**	**0.000**	−**0.05 (**−**0.10;** −**0.01)**	**0.026**	**0.17 (0.12; 0.22)**	**0.000**
	Eosinoph	−0.01 (−0.10; 0.07)	0.756	**−0.21 (−0.30; −0.11)**	**0.000**	−**0.09 (**−**0.16;** −**0.02)**	**0.008**
	Basoph	−0.04 (−0.10; 0.02)	0.203	0.03 (−0.08; 0.14)	0.575	0.00 (−0.10:0.11)	0.915
	Monoc	−0.08 (−0.17; 0.01)	0.101	**0.15 (0.05; 0.24)**	**0.003**	**0.22 (0.14; 0.31)**	**0.000**

(B) Variances (error terms)		0.92 (0.89; 0.95)		0.90 (0.85; 0.95)		0.84 (0.79; 0.89)	

(C) Covariances		**e.PTX3, e.LTL**		e.LTL, e.night-shift work		e.PTX3, e.night-shift work	

		**0.15 (0.01; 0.29)**	**0.033**	0.09 (−0.02; 0.19)	0.100	−0.09 (−0.189; 0.013)	0.089

*Bold character is displayed only for significant values*.

*Beta coefficients (Coef) with 95% confidence intervals (95%CI) and *p*-values for: (A) exogenous variables of three structural equations; (B) variances or error terms of three endogenous variables; (C) covariances from the pairwise comparison of three simultaneous equations*.

*Endogenous variables: long pentraxin 3 (PTX3: ng/ml); leukocyte telomere length (LTL: T/S); night-shift work (job done: variable yes/no). Error terms: e.PTX3; e.LTL; e.night-shift work*.

*Exogenous variables: age (years); sex (women = 0; men = 1); body mass index (BMI: kg/m^2^); waist (cm); work ability index (WAI, score: 7–49); pack-years (Py) of cigarettes; binge (≥4 UA/day); CVD (history of cardiovascular diseases hypertension included: yes = 1; no = 0); tumors (history of tumor: yes = 1; no = 0); C-reactive protein (CRP: mg/ml); Eosinoph (eosinophils: *n* × 10^3^/μl); Basoph [basophils (*n* × 10^3^/μl)]; Monoc (monocytes: *n* × 10^3^/μl)*.

In the first equation of Table 3, PTX3 values appear to decrease with increasing BMI (beta = −0.22; *p* = 0.022) and CRP (beta = −0.07; *p* = 0.000). Since “sex” was 1 for males and “CVD” was 1 for subjects reporting such disease, therefore PTX3 mean was lower in males (beta = −0.13, *p* = 0.000) and CVD (beta = −0.12; *p* = 0.000). Conversely, PTX3 level slightly increased with increasing number of pack-years smoked (beta = 0.07; *p* = 0.061). Moreover, when comorbidities that could influence inflammatory response (i.e., diabetes, tumors, and/or respiratory diseases) were included in the Charlson’s Index, PTX3 level associates positively with the Charlson’s Index (beta = 0.15; *p* = 0.005, data not shown). The proportion to which SEM fitting accounts for the variation of data (variance explained) was as low as 8% (=1 − 0.92). Therefore, most variation of this outcome should be attributed to unknown predictors.

The second equation of Table 3 shows that telomeres were shorter in subjects reporting a history of CVD (beta = −0.15; *p* = 0.000) and binge drinking (beta = −0.10; *p* = 0.004), and in those with higher values of CRP (beta = −0.05; *p* = 0.026) and peripheral eosinophils (beta = −0.21; *p* = 0.000). Conversely, the number of circulating monocytes associates with LTL (beta = 0.15; *p* = 0.003). The variance explained by the above fitting was 10% (=1 − 0.90).

Since the outcome of the third equation of Table 3 was a dichotomous variable (day and night-shift workers), the beta coefficients should be interpreted as probabilities rather than means. Thus, the likelihood of employment as night-shift worker was found to decrease with increasing age (beta = −0.13; *p* = 0.017) and BMI (beta = −0.17; *p* = 0.030), in subjects with anamnesis of CVD (beta = −0.14; *p* = 0.000) and binge drinking (beta = −0.13; *p* = 0.000), and among those with a lower number of circulating eosinophils (beta = −0.09; *p* = 0.008). Conversely, the factors that appeared to increase with night-shift working were male sex (beta = 0.10; *p* = 0.021), increasing values of WAI (beta = 0.10; *p* = 0.046), CRP (beta = 0.17; *p* = 0.000), and peripheral monocytes (beta = 0.22; *p* = 0.000). The variance explained by the above fitting was about 16% (=1 − 0.84).

**Figure 1:**
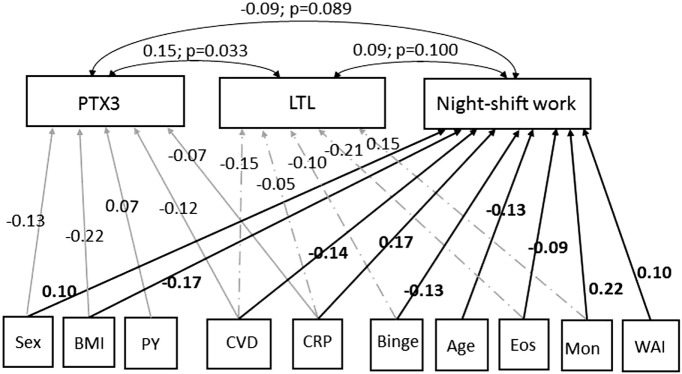
**Path diagram of the results shown in Table 3**. Square boxes stand for: endogenous variables [long pentraxin 3 (PTX3: ng/ml); leukocyte telomere length (LTL: T/S); night-shift work (job done: variable yes/no)] and for exogenous variables [age (years); sex (women = 0; men = 1); body mass index (BMI: kg/m^2^); pack-years (PY) of cigarettes; binge (≥4 UA/day); CVD (history of cardiovascular diseases hypertension included: yes = 1; no = 0); C-reactive protein (CRP: mg/ml); eosinophils (Eos: *n* × 10^3^/μl); monocytes (Mon: *n* × 10^3^/μl); work ability index (WAI, score: 7–49)]. Arrows specify the slightly significant (*p* < 0.05) causal link of variables with PTX3 (
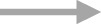
), LTL (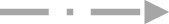), and night-shift work (→). The estimated beta coefficients appeared along the arrowed paths and the “minus” sign shows an inverse relationship. Curved routes display the cross-equation correlation of error terms resulting from the pairwise comparison of the three covariances. The estimated beta coefficients (with *p*-values) appear along the curved arrows, the “minus” sign indicates an inverse relationship.

The last row of Table 3 shows the three covariances resulting from the pairwise comparison of the three equations. The cross-equation correlation of error terms was significant (coefficient = 0.15; *p* = 0.033) comparing the first and second equations (headers: PTX3 and LTL) and in the comparison of first and third equations (headers: PTX3 and night-shift work) (coefficient = −0.09; *p* = 0.089); while it was not significant otherwise. This finding demonstrated that these variables were correlated to each other supporting the idea that increased PTX3 positively affects (protects) LTL, whereas PTX3 is slightly decreased in night-shift workers. Fitting of SEM model was evaluated with the Chi-square test (that was 0.00 with *p*-value equal to 1.00) and the stability index (that was 0.00), indicating that SEM model did not differ from a saturated model and satisfied stability condition (data not showed). The results of Table 3 were displayed as path diagram in Figure 1, using the graphical interface of SEM.

## Discussion

Our study exploring the hypothesis of an association between inflammation and biological aging, evaluated by PTX3 plasma levels and LTL in relation to night-shift work, shows by SEM analysis (cross-equation term was significant in Panel C) that PTX3 levels are positively related (protect) with LTL, whereas PTX3 levels only to some extent associates negatively with night-shift work.

The long pentraxin PTX3 is an essential component of humoral innate immunity and plays a key role in the regulation of inflammation. The innate immune response is the first line of defense against invading microbes and tissue damage. PTX3, induced by primary proinflammatory cytokines, sensing microbes, and/or tissue injury, acts as pattern recognition molecule through interaction with molecular patterns and triggers a complex response that includes the regulation of leukocyte recruitment and the modulation of complement activation (27). By interacting with provisional matrix components, PTX3 contributes to the orchestration of wound healing and tissue repair/remodeling (28). PTX3 deficiency in model mice (ApoE mice) was associated with atherosclerosis development and increased macrophage accumulation within atherosclerotic plaques as well as with more pronounced inflammatory profiles in vascular walls (17). Relative PTX3 deficiency in humans is associated with increased inflammation, cardiac damage, and atherosclerosis, while its overexpression limits carotid restenosis after angioplasty (15). On the other hand, by modulating complement-driven inflammation, PTX3 acts even as an extrinsic oncosuppressor gene (16). Global PTX3 deficiency was associated with more pronounced cancer-related inflammation and with a higher number of tumor-infiltrating macrophages. PTX3-deficient tumors were also characterized by enhanced genetic instability features as indicated by increased p53 mutations, oxidative DNA damage, and expression of DNA damage markers, in line with the hypothesis that inflammation contributes to genetic events that can lead to the genetic instability observed in tumors (29). On the whole, certain observations point toward a protective role of PTX3 against CD (15) and tumor (16), acting through a better tuning of inflammatory response (17). Therefore, the positive association of PTX3 with LTL suggests that the presence of this long pentraxin, by orchestrating an efficient governance of inflammatory processes, may protect telomere from an accelerated attrition, ensuring therefore the genetic stability of cells. Conversely, a decrease in PTX3 would not provide this such protective function.

Inspecting our sample population, it can be seen that night-shift workers were younger than day workers, and consequently with lower length of total employment. Moreover, day workers never performed night-shifts with relatively more years spent in the current job, making day workers an appropriate control group. Compared to the elder day workers, night-shift workers were physically more active and reported lesser percentages of chronic diseases, musculoskeletal disease, spinal disk hernia, and cardiovascular disease. On the other hand, an overall reduction in number of lymphocytes and monocytes found in the elder group of day workers compared to the younger night-shift worker group can be accounted to age-associated changes/effects (30). All these characteristics are reflected on a significant longer mean LTL in night-shift workers, confirming LTL measure as a reliable marker of biological aging.

In the simultaneous analysis, the first of three regressions shows that PTX3 negatively associates with BMI, CRP, and history of CVD hypertension included, suggesting that PTX3 is modulated by several aging conditions and/or disorders. In particular, the negative association of PTX3 levels with BMI (31) corroborates some of the available scanty data from literature. On the other hand, the negative relationship with CRP and CVD is in contrast with other data available (32, 33), bringing out the dual antagonistic and complex role of PTX3 in the regulation of inflammation (17). Finally, we found PTX3 to be positively associated with number of pack-years smoked. This might support the idea that cigarette smoking (CS) can be an important determinant in PTX3 production, confirming an *in vivo* study on mice (34), and that PTX3 could be an indicator of this activation. In fact, by increasing levels of PTX3, CS elicits both immune and inflammatory responses (34). Thousands of ROS are produced in each puff of the burning cigarette and are not removed by cigarette butt filters (35). CS constituents (particularly ROS and trace of microbial cell components, including bacterial lipopolysaccharide) activate epithelial cell intracellular signaling cascades that lead to inflammatory activation [e.g., interleukin-8 and tumor necrosis factor-alpha (TNF-α)] (36, 37). During inflammation, different cell types produce large amounts of PTX3 and the level of circulating PTX3 increases in several pathological conditions (38), generally correlating with disease severity (39, 40). This is in agreement with the positive relationship between PTX3 and the Charlson’s Index observed when conditions significantly impacting the inflammatory response, i.e., diabetes, tumors, and/or respiratory diseases, were included in the analysis (data not shown). The Charlson comorbidity index (24) has been widely utilized by health researchers to measure burden of disease and case mix. The index has been validated for its ability to predict mortality in various disease subgroups, including cancer, renal disease, stroke, intensive care, and liver disease (41). To our knowledge, no studies until now have investigated the relationships between PTX3 and Charlson’s Index.

The second of three regression equations confirms literature available on shorter LTL in subjects reporting a history of CVD, hypertension included (42), binge drinking (10), and in those with higher values of the CRP, the systemic inflammation marker. Inflammation (43) and the ensuing oxidative stress (12, 43), two mechanisms that accelerate telomere shortening (44), have been linked with heavy alcohol consumption (45). Essential hypertension, the most common disorder of aging and one of the leading causes of CD, is linked to oxidative stress (46), and in turn with inflammation (47). These results support the hypothesis that telomere attrition may be related to diseases of aging, through mechanisms involving inflammation and exposure to risk factors of CD and cancer, including hypertension and heavy alcohol drinks. An increase in eosinophils in relation with shorter LTL, as well as with altered values of CRP, is in line with a study conducted on asthmatic subjects, in which chronic asthma (life-course-persistent asthma), via systemic eosinophilic inflammation, associated with LTL (48).

In the third regression, the likelihood of employment as night-shift worker was found to be associated with decreased age, BMI, CVD hypertension included, binge drinking, and number of circulating eosinophils. Conversely, night-shift work associates with male sex, increased values of WAI, CRP, and peripheral monocytes. These results fit well within the framework of the characteristics of our study population, wherein, among other things, night-shift workers were younger than day workers and physically more active. This is reflected in a decrease in BMI, blood pressure, eosinophils as marker of inflammation, and an increased WAI and peripheral monocytes, all features linked to younger age. On the other end, the higher CRP levels among night-shift workers, remarkably independent of the considered CD risk factors (such as smoking, BMI, and hypertension), beside confirming the results of a larger study on airline-company employes (*n* = 1,877, 60% man) (49), suggests that night-shift work is associated with increased systemic inflammation. Albeit most of our participants were female and it is known that immune/inflammatory parameters fluctuate during menstrual cycle, we missed information on menstrual cycle at the time of blood samples withdrawn. However, our female population was too heterogeneous to evaluate this issue correctly. Indeed, the number and complexity of the variables [age, phase of the menstrual cycle (follicular/luteal phase), recent pregnancies and breast-feeding, menopause, hormone medications, etc.] which influence the fluctuation of inflammatory parameters probably require an *ad hoc* study (50). Nevertheless, by SEM analysis, our work provides new evidence of inflammation as a possible pathway linking irregular nocturnal working hours and morbidity.

In conclusion, the relevant finding stemming from our work is that PTX3 positively correlates to LTL envisaging another new function of PTX3 that, by orchestrating an efficient control of the inflammatory process, may protect telomere from an accelerated attrition, ensuring therefore the genetic stability of cells. On the other end, the higher CRP levels among night-shift workers, remarkably independent of the considered CD risk factors (such as smoking, BMI, and hypertension), suggest that night-shift work is associated with increased systemic inflammation. This would make nocturnal workers more susceptible to premature aging.

## Ethics Statement

The local Ethics Committee (School of Medicine, University of Ferrara) approved the study protocol (code number 140792). All subjects gave written informed consent and the study was conducted in accordance with the Declaration of Helsinki.

## Author Contributions

All authors have a substantial contributions to the conception or design of the work; or the acquisition, analysis, or interpretation of data for the work; drafting the work or revising it critically for important intellectual content; final approval of the version to be published; agreement to be accountable for all aspects of the work in ensuring that questions related to the accuracy or integrity of any part of the work are appropriately investigated and resolved. Moreover, MB, MN, PB and MS have a substantial contribution in subject enrollment, physical examination, interview with structured questionnaires, Ethics Committee application. SP and MC in laboratory analysis; GM in statistics; BB, AM, and RL in PTX3 analysis.

## Conflict of Interest Statement

The authors declare that the research was conducted in the absence of any commercial or financial relationships that could be construed as a potential conflict of interest.
